# What ails the NIH peer review study sections and how to fix the review process of the grant applications

**DOI:** 10.20517/jca.2023.3

**Published:** 2023-01-16

**Authors:** Ali J. Marian

**Affiliations:** Center for Cardiovascular Genetics, Institute of Molecular Medicine and Department of Medicine, University of Texas Health Sciences Center at Houston, Houston, TX 77030, USA.

**Keywords:** Peer review, NIH, Study section, Grant application

The concern has been on my mind for a while, but I had procrastinated putting it down on paper till now. It was the recent announcement by the National Institutes of Health (NIH) Center for Scientific Review on the proposed changes to the review criteria of the grant applications submitted to the NIH study sections that provided the impetus to write this opinion article. The Christmas holidays provided the time needed to write it. Accordingly, “NIH proposes to reorganize the (current) five review criteria into three factors” as follows:

Factor 1: Importance of the Research.

Factor 2: Rigor and Feasibility.

Factor 3: Expertise and Resources

In the new system, the first two factors will be scored individually (from 1 to 9, with 1 being the best) and the third will not. It will be “assessed and considered in the Overall Impact Score”.

In the following sections, I will discuss the problems with the cardiovascular study sections, as I perceive them, and why the proposed changes fail to address the main problem. To begin, Factor 1 simply combines the previous two criteria, which were referred to as Significance and Innovation, into one criterion that will be named “Importance of the Research”. Notwithstanding the challenges that one faces in recognizing the “Importance of the Research”, as time has amply shown us, the change is a non-substantial one, if any. Factor 2 is what is currently called “Approach”. It will be simply a name change and nothing more. Factor 3 combines the two current criteria of Investigator and Environment as one criterion of “Expertise and Resources”. This will be the only modification when the proposed changes go into effect, as currently, the investigator and environment are scored individually. The changes are designed to de-emphasize the investigators and the institutions and score the applications based on the importance of the research and its rigor and feasibility. It is an egalitarian approach, designed likely in response to recent reports that the reviewers favor well-established investigators and major institutions. Whether there is merit to such reports is another matter and not the focus of this article. Suffice it to state that I believe the “investigator” and the flexibility in conducting research are the cruces of scientific discoveries. The impact of empowering the investigators is illustrated in the finding that the Howard Hughes Medical Institute investigators, who are well-established investigators with ample flexibility in conducting their research programs, produced about twice more highly cited articles than the comparable investigators at the NIH but with lesser flexibility^[1]^.

NIH recently “issued a request for information (RFI) seeking feedback on revising and simplifying the peer review framework for research project grant applications.” I wrote brief feedback and received acknowledgment of its receipt but never saw it posted on the designated blog. Given that I am deeply concerned, not much about the proposed revisions of the review criteria but with the gradual changes that have occurred over the last decade or so, I could not help but pen my opinion. I am afraid those in charge of the NIH Center for Scientific Review have made the wrong diagnosis about the ailment of the grant review system over the last several years and, unfortunately, continue to do so. Perhaps, this reflects the dissociation of the decision makers from science and their understanding of it from the so-called “ivory towers”.

So, what ails the NIH study sections in charge of reviewing the grant applications? The answer is their compositions. In the current structure, a Scientific Review Officer (SRO), typically a scientist with an advanced degree, who has been away from the bench research for years if not decades and is naturally not fully aware of the reviewer’s scientific attributes, selects the reviewers to compose the study section. I assume the SROs identify the reviewers based on their publication records while focusing on the diversity of the panel, in all aspects - in accord with the current trend in society. Identifying and recruiting reviewers with the proper expertise to match the diverse pool of applications is a daunting task. The challenge is amply evident for the editors who are involved in the peer review of papers submitted to scientific journals. The process requires much more in-depth knowledge of the candidate reviewer’s aptitude that could be garnered by searching public databases and glancing at the title of their publications. I contend that it is almost impossible for a single SRO to find proper reviewers for the diverse pool of applications that are assigned to each study section. This flawed approach is reflected in the composition of the study sections, which include reviewers who are mismatched to the scientific contents of the applications they are assigned to review. It is not too infrequent that a study section member acknowledges a lack of expertise in the scientific area of the assigned application, and yet, in the current system, such an applicant is empowered to “kill” the application.

To support my contention, I have reviewed the performance metrics of the reviewers who served during the last cycle (October 2022) in five major NIH study sections in the cardiovascular field. The study sections are the Clinical Integrative Cardiovascular and Hematological Sciences (CCHS), Cardiovascular Differentiation and Development (CDD), Integrative Myocardial Physiology/Pathophysiology A (MPPA), Integrative Myocardial Physiology/Pathophysiology B (MPPB), and Therapeutic Development and Preclinical Studies (TDPS). I have chosen the publication metrics to analyze the scientific expertise of the study section members, as, despite their shortcomings, such indices provide the most objective criteria and, by and large, are useful tools in assessing the scientific contributions of the investigators. The source of the metrics is the Web of Science by Clarivate, which is likely the most authoritative and comprehensive database for this purpose. It is the database that is used to calculate the infamous impact factor of scientific journals.

I have included the data for 136 reviewers except for 3, whose identity I could not ascertain with reasonable confidence (reviewers with the same or very similar names). Whenever ambiguous, I used the current institutional affiliation to identify those who did not have a unique name and then searched to include all their articles. The data are not perfect and likely have missing components. For example, some authors might have listed their names in different ways in different articles, and therefore, I might have missed captured some of their articles. None of the indices that I have selected is perfect, as there is none. It is best to assess the content of the scientific discoveries, but that is not a feasible task for this purpose. Of course, researchers are typically informed about the scientific contributions of the investigators in their corresponding fields, a privilege that is not available to the SROs. Overall, the selected indices provide useful information that informs about the scientific qualifications of the reviewers who served in the last cycle of the study section:

1. H index. It is the number of articles published by a reviewer which have been cited at least the same number of times. The H index, per Jorge E. Hirsch, a physicist who designed it “is an estimate of the importance, significance, and broad impact of a scientist’s cumulative research contributions”^[2]^. It is less susceptible to outliers, in contrast to some other indices that use the mean values.
2. The number of articles published by each reviewer. The category, by and large, means the original research articles. It excludes, albeit imperfectly, the review articles, meeting abstracts, editorial material, and others. Unfortunately, it does not exclude some of the articles that compile statistical data on the burden of cardiovascular diseases, such as the annual report on “heart disease and stroke statistics”. It is an indicator of the scientific output of a reviewer.
3. The total number of citations to all articles published by the reviewer (times cited). This is a representation of the influence of the reviewer’s articles in the scientific community.
4. The average number of citations per article published by the reviewer. This is simply the total number of citations divided by the number of articles published by the reviewer. It is an indicator of whether the reviewer generally publishes influential articles.
5. Citations to the reviewer’s article that resides in the median of articles published by the author. This index is less subject to outliers, which could change the mean values markedly.
6. Citation number to each reviewer’s top-cited article.
7. The average number of citations per year to all articles published by each reviewer. This index also reflects the overall influence of the reviewer in the scientific community.

The indices used in this analysis, except for the H index and the median values, are highly susceptible to outliers. Two notable outliers are co-authors in the articles that report on heart disease and stroke statistics and the global burden of disease. Such articles commonly receive a very large number of citations. They are major contributors to the impact factors of the journals that publish them and, likewise, the metrics of the involved authors. Thus, the data are presented with and without the two major outliers.

Among the selected variables, the H index is less susceptible to outliers^[2]^. The distribution plots of the H index for all reviewers and after the removal of the two outliers are shown in Figures 1 and 2. The mean ± SD of the H index of all reviewers (*N* = 133) was 22.5 ± 11.9 and the median was 20 [Table 1]. The range varied from 2 to 75, with 69 reviewers (51.5%) having an H index of 20 or less. Except for a few, which included the outliers, almost all reviewers had an H index of less than 40. While there is no clear cut-off point, an H index of ~40 or higher, in my opinion, denotes an experienced investigator. An H index of less than 20, in the opinion of many, indicates inadequate scientific experience. Fourteen reviewers had an H index of 10 or less. Such reviewers are considered neophytes in terms of scientific experience. I recognize that among this low H index group, there may be brilliant young scientists with an incredible ability to see the forest for the trees. In general, such wisdom and judgment typically come from years of scientific explorations and experience.

The distribution plots of other indices used to assess the scientific productivity of the members of the study sections are shown in Figures 1 and 2. The data, by and large, point to a paucity of experienced investigators in the study sections. To avoid redundancy, as the data are presented in the tables and figures, only a couple of additional indices are briefly discussed. The number of original research articles published, despite its limitations, is a good indicator of the scientific productivity of an investigator. I recognize that the quality and content are the true scientific contributions and not the number of papers [please see my Podcast (Available from: https://cardiovascularaging.com/podcasts/view/10)]. However, quality and content are much harder to judge and quantify. Not knowing the true scientific contributions of the authors is one reason that SROs are not in the best position to select the proper reviewers. The mean and median numbers of the articles published by the 133 study section members were 58 and 42, respectively, which are modest for a scientific panel. There were close to two dozen reviewers who had published 20 articles, whereas there were less than 20 reviewers who had published more than 100 articles. Citations on the investigator’s work also serve as an indicator of the impact of the investigator’s research findings in the scientific field, albeit like most metrics, it has serious shortcomings. On average, the articles published by the members of the 5 study sections were cited 182 times per year, with a median value of only 101.4 citations per year. These numbers are exceedingly small and suggest a modest impact in the scientific field, considering the plethora of biomedical journals (> 30,000) and the number of annual citations to articles published by top scientists is typically in the thousands.

I did not include a control group (to NIH study section reviewers), as I felt there was no proper control group. I submit that neither the editorial board members of selected cardiovascular journals nor the reviewers who served in the previous study sections years ago would be an appropriate control group. The findings of such comparisons would be subject to numerous confounders. Therefore, it is best to leave the judgment to you, the readers, and not make the assessment slave to statistical p values. Nonetheless, for those who are unfamiliar with these metrics, let us consider the case of three applicants (real people who are still applying for R01 funding). Investigator A is an established investigator who has an H index of 114 and has published 378 articles, which have been cited >45,000 times. Investigator B has published 168 articles, which have been cited > 10,000 times, and has an H index of 52. The third investigator, investigator C, has published 274 articles and has an H index of 110 and an average of 2220 citations per year. These three are established investigators who have made major discoveries but are not the top-cited scientists in cardiovascular research. Would you consider someone with an H index of 20, who has published 42 articles, which have garnered a total citation of ~1500 (all numbers are the median values of the study section members), to be a peer to investigator A, B, or C? Would a member of the study section who has an H index of < 10 and has published < 10 original research articles be qualified to review a grant application by investigator A, B, or C? It seems that only a few in the above 5 study sections have the scientific experience to match the scientific accomplishments of investigators A, B, and C and hence, serve as a peer. Overall, the scientific accomplishments of an average member of the 5 study sections are less sparking than those of an average established cardiovascular scientist, who typically has an H index of > 40 and has published over 80 articles, which have garnered over 8000 citations.

The data, in my opinion, demonstrate the shortcoming of the study sections, which are comprised, in part, of inadequately experienced reviewers. Considering that the current format enables one reviewer, no matter how inexperienced, who is unenthusiastic about an application to change the priority score of an application from a fundable to a non-fundable status, the current state is unhealthy. Science is best reviewed by the most experienced investigators who, over time, have developed the insight necessary to identify the best scientific programs from those which might be ostentatious and therefore, more attractive to inexperienced reviewers. Such flashy research projects typically fit the category of “improbable and implausible” and produce irreproducible results^[3]^.

Considering the data shown in Tables 1 and 2 and Figures 1 and 2, I am afraid that those at the helm of the NIH Center for Scientific Review are leading us up in a blind alley. The proposed remedy of changing the review criteria from five to three is off-target and is expected to be ineffective in improving the peer-review system. Thus, I propose the followings, which might be considered too radical from the current approach, but they are simple and necessary, in my opinion, to improve the current review system at the NIH study sections:

1. Change the function of the SROs. The SROs, for obvious reasons discussed above, are not in the best position to identify and select reviewers for the grant applications. Selecting proper reviewers is a very arduous task. It requires a good understanding of the reviewers’ scientific expertise, the robustness of their scientific findings, and their dedication to a fair and just peer-review process. The main function of each SRO should be to identify and recruit co-chairs of the study section and leave it to the co-chairs to identify and recruit the reviewers (further below). The function of an SRO will be analogous to the function of the managing editor of a scientific journal, while the chairs function as the editors.
2. Recruit three or more senior established investigators to function as co-chairs. I emphasize senior investigators who have been around long enough to see the forest for the trees. Three is not the magical number, but one chair is insufficient to select the proper reviewers to match the diverse science proposed in the applications. The co-chairs will identify and recruit the reviewers according to the scientific contents of the applications, like journal editors who identify the expert reviewers, after reviewing the content of the submitted papers. Reviewers will be selected based on their scientific expertise, regardless of their academic ranks and other criteria, but must be experienced ones.
3. Empower the co-chairs to rectify errors. The co-chairs must be empowered to bring up for discussion any comments of the reviewers that might be considered scientifically incorrect or a misunderstanding. Currently, the chairs are not expected to intervene as such interference is considered an undue influence on the study section members, because of the status associated with the chair position. It is important, however, for the chairs to be active and bring up for discussion the comments that they might consider erroneous or a misunderstanding. Unless the erroneous comments are discussed and if necessary, corrected, they could have a drastic false influence on the priority scoring by the entire study section. Having three co-chairs or more will reduce the chance of one co-chair exerting an excess influence.

The above approach or a similar one that emphasizes the role of experienced reviewers in judging the scientific merit of the applications is practical and essential for funding the most meritorious grant applications. I spent hours collecting the data for 133 reviewers, as I sincerely worry about the current state of the grant application review process at the study sections and therefore, its impact on the state of funding of cardiovascular sciences in the US. I worry that those in charge have missed the diagnosis. I feel obliged to raise the concern, even though many are and have been aware of the problems but prefer to remain silent. I am hopeful that this commentary will provoke a serious and sincere discussion on this very important topic, which determines the future of cardiovascular sciences in the US and hence, the lives of many worldwide.

In summary, the problem is not the review criteria. The problem is the composition of the study sections, which include inadequately experienced reviewers. To enhance the outcome of the peer review of the grant applications and to fund the best science in the US, the leadership of the NIH Center for Scientific Review needs to focus on the structure of the study sections. Otherwise, there is a substantial risk of not funding the best science, which would hamper the discoveries in cardiovascular sciences and hence, the cure of cardiovascular diseases.

## Figures and Tables

**Figure 1.**
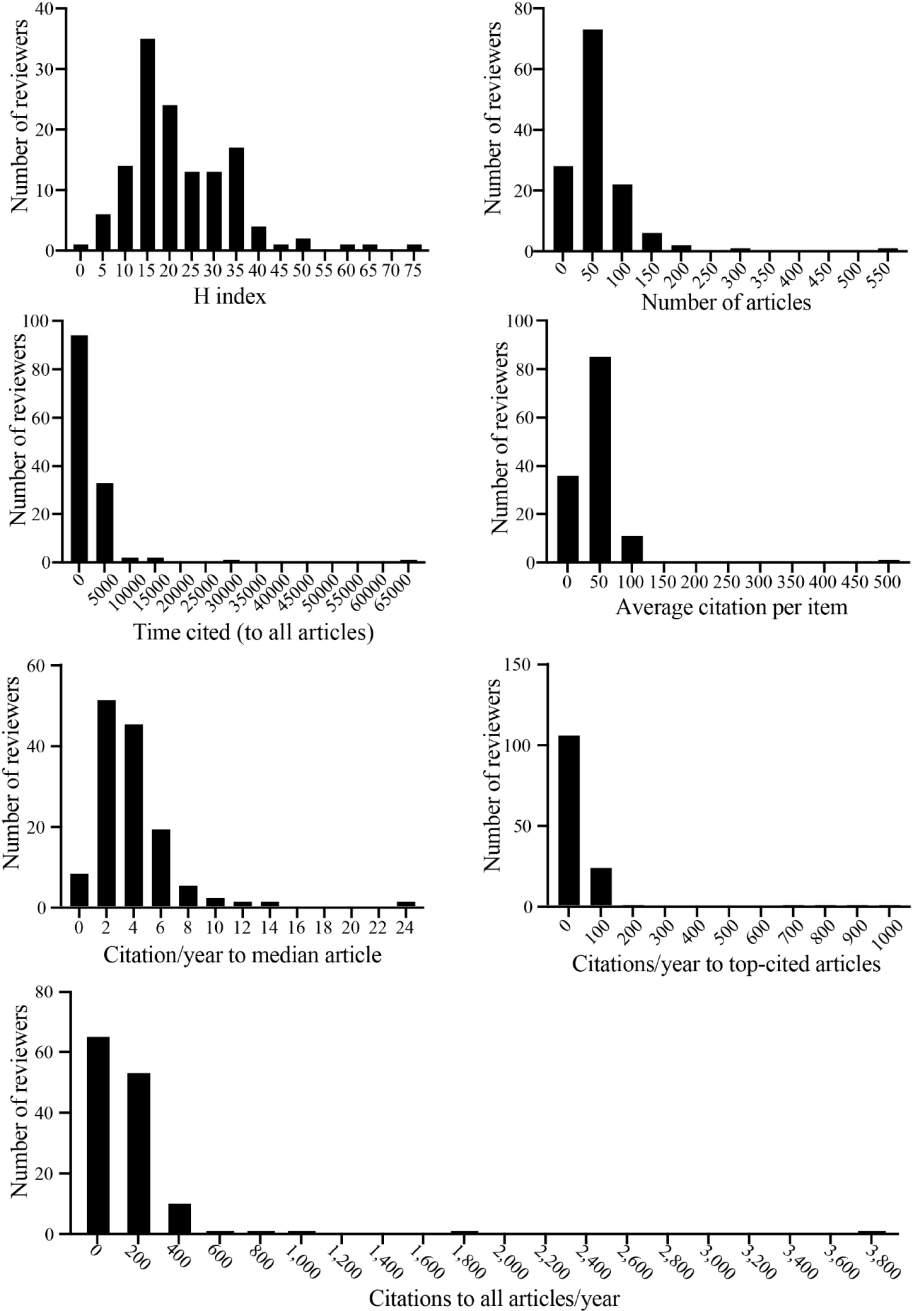
Distribution plots of 7 indices that were used to analyze the performance metrics of 133 reviewers who served in the Oct 2022 cycle of the 5 cardiovascular study sections

**Figure 2.**
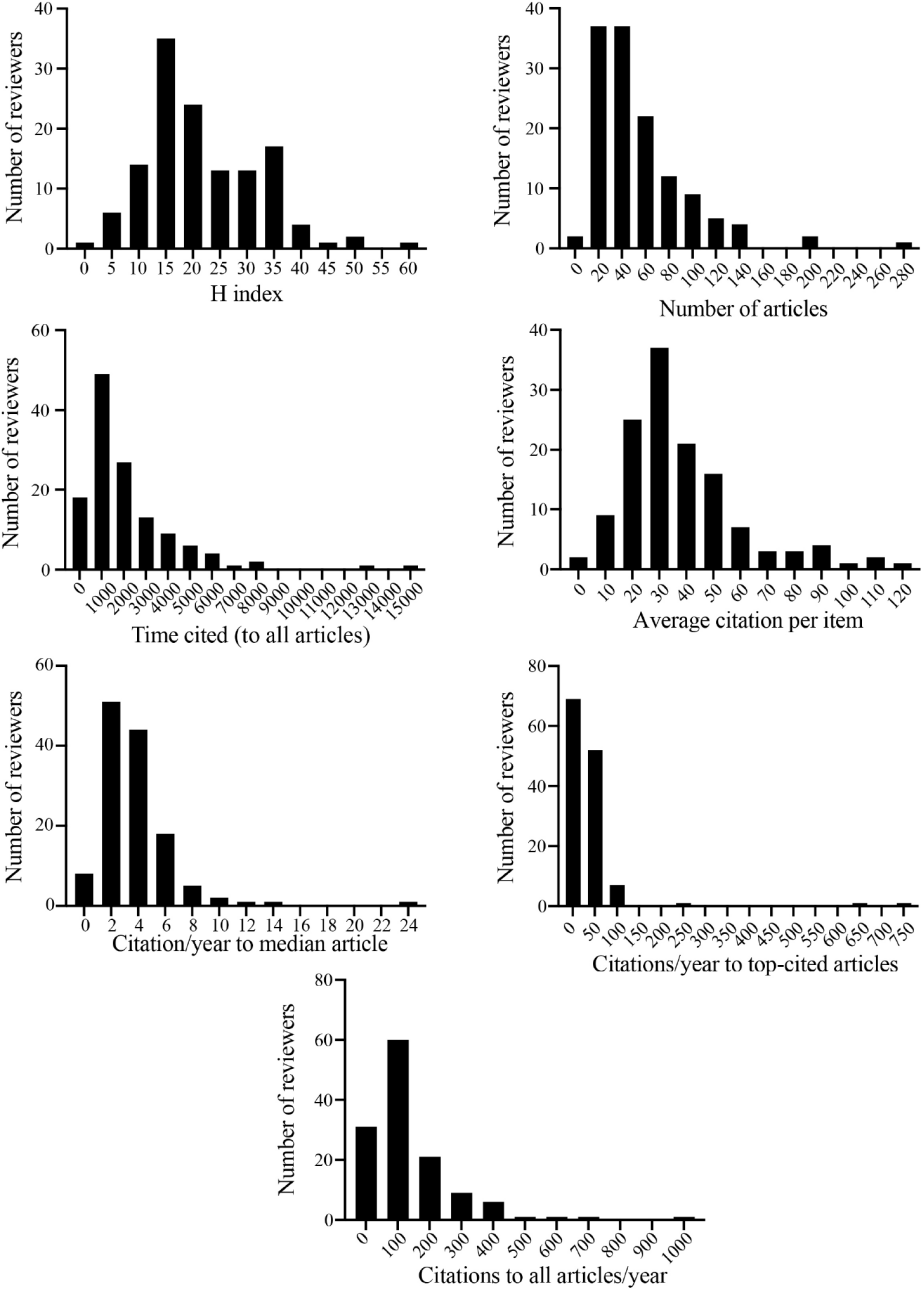
As in Figure 1 but after the exclusion of the two reviewers who were outliers.

**Table 1.** Performance metrics of five NIH cardiovascular study section reviewers

	Mean ± SD	Median	95%CI	Minimum	Maximum

N	133	133	133	133	133
H index	22.5 ± 11.9	20	20.4–24.5	2	75
Number of articles	58.5 ± 58.9	42	48.4–68.6	3	531
Times cited	2867.1 ± 6351.5	1493	1777.6–3956.5	12	64,369*
Average citations per item	41.4 ± 45.1	33.3	33.7–49.1	2.3	491.4
Citation to median article/year	3.7 ± 2.8	3.2	3.2–4.2	0.4	24
Citations/year to top-cited articles	54.3 ± 140.1	23.3	30.2–78.3	0.6	988*
Citations to all articles/year	182.1 ± 374.8	101.4	117.9–246.1	1.6	3786*

* The numbers represent citations to the American Heart Association’s “Heart Disease and Stroke Statistics” and “Global Burden of Disease” articles. The articles are outliers in the dataset.

**Table 2.** Performance metrics of five NIH cardiovascular study section reviewers after removal of two outliers

	Mean ± SD	Median	95%CI	Minimum	Maximum

N	131	131	131	131	131
H index	21.7 ± 10.3	20	19.9–23.5	2	60
Number of articles	54.4 ± 41.8	42	47.1–618.6	3	283
Times cited	2184.3 ± 2321.3	1467	1783.0–2585.5	12	15,327
Average citations per item	37.8 ± 22.3	33.0	34.0–41.7	2.3	116.1
Citation to median article/year	3.7 ± 2.9	3.1	3.2–4.2	0.4	24
Citations/year to top-cited articles	40.8 ± 88.4	22.8	25.6–56.1	0.6	750.2
Citations to all articles/year	142.0 ± 140.2	100.2	117.8–166.3	1.6	957.9

Data after removal of the American Heart Association “Heart Disease and Stroke Statistics” and “Global Burden of Disease” articles.

## Data Availability

Not applicable.
